# Ambidextrous human resource practices and individual creativity—A cross-layer multi-time analysis based on psychological capital and QLMX

**DOI:** 10.3389/fpsyg.2022.944616

**Published:** 2022-11-11

**Authors:** Fuqiang Zhao, Longdong Wang, Yun Chen, Wei Hu

**Affiliations:** ^1^School of Management, Wuhan University of Technology, Wuhan, China; ^2^School of Management, Jiangsu University, Zhenjiang, China

**Keywords:** ambidextrous human resource practices, quality of leader-member exchange, psychological capital, individual creativity, social exchange theory, resource conservation theory

## Abstract

As an important factor in enhancing individual creativity, employees’ psychological capital has been widely tested by scholars. However, the effects of ambidextrous human resource practices (AHRP) on individual creativity has not been confirmed. On the basis of the theories of social exchange and resource preservation, we explored the mechanism of AHRP’s impact on individual creativity using three-point data collected from March to September 2021 from 23 large enterprises in the service, finance, construction, and education sectors in five Chinese cities: Wuhan, Shanghai, Guangdong, Shenzhen, and Zhengzhou. A cross-layer regression and a Monte Carlo sampling analysis were performed on 135 pairs of leaders and 810 employees. In addition, we tested the cross-layer mediating effect of the psychological capital between AHRP and individual creativity and the boundary effect of the quality of leader–member exchange (QLMX) in the cross-layer effect. Research results indicate that (1) AHRP have positive effects on individual creativity across layers; (2) psychological capital mediates the cross-layer relationship between AHRP and individual creativity; (3) QLMX moderates the direct effect of AHRP on employees’ psychological capital; and (4) QLMX moderates the indirect effect of AHRP on individual creativity through psychological capital. The research conclusions lay a theoretical foundation for AHRP at the organizational level and provide a guiding reference for the enhancement of employee creativity at the individual level.

## Introduction

In the VUCA (Volatility, Uncertainty, Complexity, and Ambiguity) era, organizational innovation is the main approach used to maintain the survival of an enterprise; it is the core force to promote team development and an important cornerstone to lay the organization forward (Tierney et al., 1999). Organizational innovation comes from the individual creativity of employees (Van and LePine, 1998; Zhou and George, 2001). Therefore, individual creativity has become an important driving force for organizational development. Existing studies have shown that human resource practices can affect employees’ innovation motivation (Kianto et al., 2017), innovative behavior (Gu et al., 2015), and innovation performance (Kianto et al., 2017). Moreover, different types of human resource practice combination have cumulative synergistic effects (Gong et al., 2009). In the process of employees pursuing utilization and exploratory innovation, a single-oriented human resource practice can no longer meet the needs of dual innovation. Therefore, organizations not only need to use internal resources to achieve incremental innovation, but also through the acquisition of external resources to achieve radical innovation, the integration of internal and external resources to meet the needs of individual dual innovation and organizational dual development (Devanna and Tichy, 1990). Ambidextrous human resource practices (AHRP) have been proposed in response to the call to solve the problem of innovation paradox with a dialectical and unified view of balance; incorporate commitment- and cooperation-oriented organizational innovation into the management thinking framework of human resource practices while coordinating and balancing the dual innovation activities of enterprises; maintain the dynamic competitive advantage of enterprises (Bledow et al., 2009); and enhance the dual creation of employees’ force.

Existing studies have shown that commitment-oriented human resource practice (CM-HRP) is conducive to the utilization of redundant knowledge within the organization to achieve incremental innovation, while cooperation-oriented human resource practice (CO-HRP) helps the organization to input non-redundant external knowledge to promote radical innovation (Anand et al., 2002). Therefore, AHRP is different from other types of human resource practices, which can significantly improve individual creativity (Chen et al., 2021).

Psychological capital is a manifestation of employees’ psychological state or traits, including confidence in success, optimism about attribution, hope for goals, and resilience to adversity (Karatepe and Karadas, 2014). Conservation of resource theory points out that psychological capital, as an individual’s positive mental state, positively affects employees’ attitudes, behaviors, and performance (Luthans et al., 2005). Innovation requires the courage to break conventions and self-affirmation. Employees who lack self-confidence need the courage to make breakthroughs in innovative thinking. A higher sense of psychological security drives the employees to have stronger willingness to innovate and participate, which can also increase their creativity (Baer and Frese, 2003). Psychological capital has an important internal transmission mechanism in the process of “management practice—mental state—behavior result.” Therefore, this study introduces psychological capital to examine its mediating role between AHRP and individual creativity.

On the basis of social exchange theory, although the human resource practices implemented by an organization can affect the attitude, behavior, and performance of employees, their effective role depends on the quality of the exchange relationship between employees and leader (He et al., 2015). Affected by individual resource constraints and personality preferences, leaders cannot maintain equal relationship exchanges with each team member, and such exchanges can be varied, which leads to differences in the quality of leader–member exchange (Regts et al., 2019). Social exchange theory indicates that QLMX can bring different psychological capital and behavioral manifestations to employees (Cropanzano and Mitchell, 2005). High QLMX can provide employees with different resource tilts, which can bring them higher work performance (Regts et al., 2019) and organizational citizenship behavior (Kacmar et al., 2011; Chughtai, 2014). Therefore, QLMX plays an important role in the formation of individual creativity (Tu and Lu, 2013; Chughtai, 2014; Gu et al., 2015; Tu et al., 2018). In line with this notion, we introduce QLMX into the research framework to test the boundary effect among AHRP, psychological resources, and individual creativity.

This study has three contributions. First, this study enriches the research on AHRP, including the investigation of the effects of AHRP the team level (Prieto and Pilar Pérez Santana, 2012) and its trickle-down effect within the team (Hu et al., 2017). Our study also enriches the research on AHRP from the perspective of individual psychological capital and provides individual-level reference for future research. Second, our work enriches the research on the relationship between leaders and members with high power distance in the Chinese context. QLMX has different effects in various cultural situations (Han and Yang, 2011). Under the high power distance in China, QLMX can affect the leadership style, team atmosphere, and organizational human resource practices (Chen et al., 2007). Given that this study is conducted in the Chinese context, our study enriches the exchange of quality literature by leaders. In addition, it makes a further comparison with the relationship between leaders and members in the existing Western cultural context and lays a contextual basis for future research. Finally, this study further verifies the application of social exchange theory in organizational situations, extends it to the organizational atmosphere of AHRP, and makes a theoretical contribution to the further improvement of social exchange theory.

In summary, this study is based on social exchange and resource conservation theories. It aims to reveal the black box mechanism of AHRP’ effects on employees’ individual creativity and examine the mediating role of psychological capital in this relationship and the boundary effect of QLMX.

## Theoretical background and hypothesis development

Human resource practices are a specific method of business management, and the connotation of different types of such practices may vary; however, the core influence mechanism is essentially the same (Oldham and Cummings, 1996). Specifically, commitment–cooperation-oriented AHRP also influence the output of innovation results by managing employees’ work ability, motivation, and opportunities (AMO; Colakoglu et al., 2006). In view of the ambidextrous system perspective, AHRP deal with the ambidexterity of organizations; use paradox thinking to meet the innovation needs of organizations and individuals; address the contradictions and tensions existing in organizations; and implement a compound human resource management practice, which ensure the coordination and integration of organizational resources to promote dual innovation. With reference to existing research and the AMO paradigm of human resource management, the implementation of commitment–cooperation-oriented AHRP within organizations can provide contextual assistance for the organizations’ utilization and exploratory innovations (Xiao, 2018). Commitment-oriented human resource practices are a series of practice collections that promote the improvement of employees’ skills, provide employees with growth opportunities, and endow employees with knowledge and skill protection for participating in innovative behaviors. With the goal of improving work efficiency, commitment-oriented human resource practices promote employees’ utilization-based innovation level. Conversely, cooperation-oriented human resource practices are a collection of practices that improve employees’ cooperation ability, stimulate cooperation motivation, and provide cooperation opportunities. These practices are oriented to work upgrades and reforms and promote employees’ exploratory innovation level (Chang et al., 2011). Existing empirical studies have confirmed that the implementation of the organizational context of commitment-oriented human resource practices is helpful for internally refined knowledge management to achieve innovation, whereas the implementation of the organizational context of cooperation-oriented human resource practices can help find the input of external nonredundant knowledge to promote innovation (Anand et al., 2002). Therefore, in view of the ambidextrous perspective of organizations, the AHRP of commitment–cooperation orientation have important research value for exploring the paradox integration mechanism in organizations.

The psychological capital of employees is their perception of the possible consequences of their behaviors in the work environment (Newman et al., 2018). Economists regard psychological capital as a relatively stable psychological tendency or characteristic formed by individuals in their early life. For example, Luthans et al. (2005) claimed that psychological capital refers to people’s positive mental abilities; and they listed self-confidence/self-efficacy, hope, optimism, and resilience according to the POB standard (measurable, developable, and can be used to improve job performance). They distinguished psychological capital from self-esteem, self-evaluation, emotional intelligence, and positive psychological traits based on the POB standard. Inside and outside the workplace, individual psychological capital has a positive effect. Using the general public as a sample, psychologists have found that optimism and hope have significantly positive effects on personal health, adaptability and adjustment ability, stress reduction, planning behavior, and employment (Luthans et al., 2007). In the workplace, scholars have found that the overall construction of psychological capital or individual elements promote individual job satisfaction (Karatepe and Karadas, 2015), organizational commitment (Luthans et al., 2005). The overall construction of psychological capital or individual elements also reduce the absentee rate of subordinates; promote the effectiveness of organizational change; increase the number of established companies; and improve organizational resilience, profitability, and company performance. Numerous research conclusions have laid a literature foundation for us to conduct our study on AHRP in the organizational field to improve psychological capital.

Leading-member exchange is the exchange relationship between leaders and members in an organization. Due to the limited resources, the general leader may establish a differentiated exchange relationship with his members, and thus adopt a differentiated strategy to treat members inside and outside the circle (Wayne et al., 1997). Based on the theory of social exchange, as informal organizational support, leaders’ support for employees includes emotional and tool support or creativity, work and social support, and their behavior positively affects employee behavior (Tekleab and Chiaburu, 2011), and leadership support perception promotes employee performance (López-Cabarcos et al., 2022). High QLMX allows members in the circle to obtain higher degrees of freedom, greater decision-making power, and more organizational support (Bauer and Green, 1996). Due to the limited information and resources of leaders, leaders treat different subordinate members in different ways and strategies. They tend to treat “in-the-circle” members through informal rules, emotions, trust, and relationships of social exchange; and through the formal authority of economic exchanges, contract rules and formal policies to treat “outside the circle” members (Han and Yang, 2011). Therefore, QLMX has become an important boundary that affects individual psychology and behavior in the organization.

### AHRP and individual creativity

In the era of cross-border integration with increasingly turbulent environment, organizations need to balance current and future development, so it is necessary to implement dual innovation compatibility and achieve the match between organizational strategy and external environment (Sun et al., 2018). Employee creativity refers to the new ideas, ideas, products, services or processes generated in the work, and is also the key to the realization of organizational dual innovation. However, the creativity needs more time consumption, cognitive effort and divergent thinking, so it needs the support of physiological, psychological, social and organizational resources (Shalley and Gilson, 2004).

Social exchange theory poses those employees pay special attention to the resources obtained and contributed by organizations and the interaction among the organizations’ members. When the human resource management practices implemented by the organization are perceived by employees as the organization’s appreciation, recognition, and investment, the organization and its employees can form a social exchange relationship rather than a purely economic relationship (Zhong et al., 2016). Human resource practices focus on experience and background when recruiting and selecting employees who are more in line with the corporate values and culture of an organization, thereby providing preconditions for employees to develop their creativity. On the one hand, commitment-oriented human resource practices are conducive to identifying employees with creative potential, high matching of people and positions, and improving the internal consistency of employees’ creativity; on the other hand, cooperation-oriented human resource practices have wide flexibility, provide numerous external resources, attract more talents with different social backgrounds and multiple knowledge systems, play the role of external social networks, and promote the creativity of employees (Wang et al., 2015).

Innovative activities not only require employees to have certain skills, motivations and opportunities, but also a fault-tolerant working atmosphere. Research has found that a human resource work system composed of a series of mutually reinforcing and cooperating human resource practices can bring higher innovation to the enterprise performance (Laursen and Foss, 2003). The first dimension of AHRP: CM-HRP not only helps to provide employees with growth opportunities, promotes the improvement of employee skills, and provides knowledge and skills protection for the generation of employees’ innovative behaviors. In addition, a safe working atmosphere is formed in the organization to reduce the perceived risk of employees’ innovative behaviors, and thus actively participate in knowledge sharing and innovation activities (Collins and Smith, 2006), which is conducive to improving their innovation performance. CM-HRP enhances employees’ sense of security and fairness by meeting employees’ basic needs for equal rights and job guarantees, thereby increasing employee organizational trust (Chen et al., 2004). Through the internalization of shared values, employees can be promoted to carry out utilization innovation activities in a more reasonable working mode, which can offset or weaken employees’ sense of resource exhaustion caused by the fact that a large amount of external heterogeneous knowledge and information cannot be effectively coordinated and integrated with the internal knowledge reserve of the organization (Truss et al., 1997).

The second dimension of AHRP: CO-HRP can enhance employees’ external cooperation ability, stimulate external cooperation motivation and provide external cooperation opportunities, thereby promoting the flow and utilization of internal and external resources and information in the organization (Wang et al., 2015). CO-HRP’s flexible work design and diversified communication channels help organizational members establish cross-departmental and cross-organizational social networks, provide more opportunities to obtain heterogeneous information and knowledge, and help employees identify and grasp innovation opportunities (Evans and Davis, 2005); its training and development can effectively improve employees’ ability to recognize the value of new knowledge, and bring benefits to the organization by digesting, absorbing and applying external new knowledge (Cohen and Levinthal, 1990). CO-HRP can promote the exchange of knowledge and information between the organization and external personnel or institutions. While improving the organization’s knowledge absorption capacity and stimulating knowledge creation to promote organizational exploratory innovation, it can offset the organizational inertia caused by CM-HRP’s over-emphasis on job support and creating a safe atmosphere, and make up for the lack of job motivation and initiative triggered by employees’ over-commitment (Sinclair et al., 2005).

Therefore, AHRP’ complementary and synergistic mechanisms jointly influence employee behaviors to meet the employees’ multi-level work and psychological needs at the same time and provide contextual assistance for improving employee innovation performance (Liu et al., 2017). On this basis, we propose the following hypothesis:

*Hypothesis 1*: AHRP has a positive impact on individual creativity.

### The mediating role of psychological capital

The psychological capital of employees is a manifestation of employees’ psychological state or traits, including confidence in success, optimism about attribution, hope for goals, and resilience to adversity (Karatepe and Karadas, 2014). Psychological capital is a holistic construct. In comparison with other positive mindsets, the core concept of psychological capital is that it has a similar state and is highly developable. It exhibits the positive psychological state of individuals and can affect the behavior, attitude, and performance of employees. From an individual level, psychological capital comes from the process of growth and development. It is a highly positive psychological state, which mainly includes optimism, self-confidence, resilience, and hope. It can have an important effect on individuals’ work attitude, cognitive style, and behavior ability and can bring a positive effect on the communication and cooperation among employees (Zhao et al., 2019).

Existing studies have found that psychological capital can play an important predictive role in individual positive behaviors (Agarwal and Farndale, 2017). On the basis of resource conservation theory, individuals with more resources have less risk of resource loss and can more easily obtain new resources. If employees have a positive mental state and maintain a high level of psychological capital, the organization needs to provide more resources to achieve the spiral of value-added resources. Employees with high levels of psychological capital often have greater performance output than employees with low levels of psychological capital (Luthans et al., 2005). When individuals are limited by their own resources, employees with high levels of psychological capital tend to respond actively in the process of resource allocation, thereby effectively resolving individuals’ problems caused by insufficient resources. As an individual’s positive mental state, psychological capital can have a positive effect on employees’ attitudes, behaviors, and performance (Luthans et al., 2005). On this basis, this study proposes the following hypothesis.

*Hypothesis 2*: Psychological capital mediates the relationship between AHRP and individual creativity.

### The moderating role of QLMX

Although the practice of human resources affects the attitude, behavior, and performance of employees, the effective play of its role depends on QLMX. Affected by individual resource constraints and personality preferences, leaders cannot maintain equal relationship exchanges with each team member and may be different, which leads to differences in QLMX (Gardner et al., 2019). As the spokesperson of the organization, leaders in the organization influence the interpretation, evaluation, and use of human resource practices by employees. In the investigation of leader–member relations, scholars have often analyzed and summarized interpersonal communication in social life and work environment based on social exchange theory; they believe that if one party obtains certain resources from the other party, then it can willingly give back and give back through attitude, emotion, and behavior (Agarwal and Farndale, 2017). High QLMX provides employees with a trust and respectful working atmosphere, which is a favorable environmental resource. To generate resource increments, employees can be more actively involved in work and study. The leader–member exchange theory believes that the relationship between leaders and employees is a highly typical social exchange relationship (Regts et al., 2019). When QLMX is high, leaders tend to trust and care more about employees, reward them, and give them more promotion space. Moreover, employees can be willing to give back and hope to continue to maintain such an exchange relationship. A large number of research results have shown that if leaders and members can maintain a high-quality exchange relationship, then employees’ attitudes and behaviors can change positively (Agarwal and Farndale, 2017).

The effect of high QLMX moderates the effects of AHRP on individual creativity, which develops employees’ ability to explore external resources and utilize and allocate internal resources more rationally, encourages employees to share knowledge, and obtains feedback from the organization in time, thereby improving employees’ hope and confidence in dealing with work difficulties (Zhao et al., 2019). High QLMX makes it easier to build trust between employees and leaders, allowing employees to understand the development of the organization and obtain development opportunities. Leader–member exchange can make employees have more empowerment. As a means of motivation (Zhou and George, 2001), empowerment can enhance employees’ confidence and resilience, thereby promoting individual creativity. On this basis, we propose the following hypothesis.

*Hypothesis 3*: QLMX promotes the transformation of AHRP into psychological capital. The higher the level of QLMX, the stronger this promotion relationship.

### Conditional process model

In summary, AHRP have positive effects on individual creativity, which can be mediated by individuals’ psychological capital. As a boundary condition, QLMX not only moderates the direct influence of AHRP on individual creativity but also moderates the indirect influence of AHRP on individual creativity through psychological capital. On this basis, we propose the following hypothesis.

*Hypothesis 4*: QLMX moderates the mediating role of psychological capital between AHRP and individual creativity. The higher the level of QLMX, the stronger the mediating relationship.

**Figure 1:**
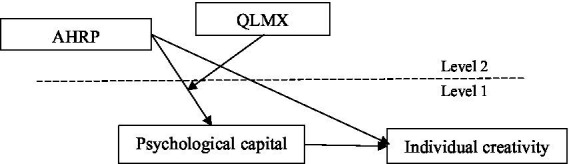
Research model.

In summary, the research model is shown in Figure 1.

## Materials and methods

### Participants and procedures

In order to reduce the common method bias, this study used multi-temporal data, and the sample distribution included Beijing, Shanghai, Guangzhou, Wuhan, and other cities. The data selected middle- and high-level managers and their direct employees as the research objects. The AHRP, QLMX, and psychological capital scale were filled out by employees. Given that employees can intuitively feel the effects of human resource practices and their emotional status changes, individual creativity and questionnaires were evaluated by managers, because managers have a more objective evaluation of the degree of employee innovation. To accurately reflect the causal relationship between AHRP and individual creativity, this study measured at multiple points in time. At T1, demographic variables, QLMX, and AHRP were measured; at T2 (after 3 weeks), employees’ psychological capital was measured; and at T3 (after 3 weeks), individual creativity was measured. The questionnaires were distributed by on-site distribution and were recycled subsequently. To ensure the authenticity of the study, the questionnaires were filled out anonymously, and the purpose of the research was fully explained at the beginning of the questionnaire. The respondents were promised that the data would only be used for academic research and would be treated confidentially. In this study, 135 leadership questionnaires and 810 employee questionnaires were effectively collected. For the leadership questionnaire, 26.372% are women, 7.462% are under 30 years old, 34.175% are 30–39 years old, 44.122% are 40–49 years old, and 14.241% are over 50 years old. Undergraduate education accounted for 8.482%, undergraduate 65.886%, and postgraduate 35.632%. 82.173% are married. Service industry accounted for 21.739%, finance industry accounted for 17.391%, construction industry accounted for 34.782%, and education industry accounted for 26.086%. The term of office is 14.831% for 3–5 years, 24.312% for 6–10 years, 35.782% for 11–15 years; 25.075% for more than 15 years. Subordinate questionnaire: 33.756% are women, 38.182% are under 30 years old, 31.261% are 30 to 39, 20.245% are 40 to 49, and 10.312% are over 50. Undergraduate accounted for 10.266%, undergraduate 68.201%, postgraduate 21.533%. 56.312% are married. The term of office is 54.181% for 3–5 years, 34.128% for 6–10 years, 8.773% for 11–15 years, and 2.918% for 15 years or more.

### Measures

In order to ensure the reliability and validity of the measurement tool, this study adopted the mature scale widely used in authoritative journals at home and abroad to measure related variables. According to the cross-cultural translation-back translation procedure, through expert discussion and modification and optimization, we strove to achieve accurate semantic expression, standard expression form, and conform to Chinese language standards, and finally finalize the formal questionnaire. Unless otherwise specified, all scale items were measured using the Likert 5-point scale method. 1 represents “strongly disagree” and 5 represents “strongly agree.”

### Ambidexterity human resource practice

Measured using the Ambidexterity human resource practice (AHRP) scale developed by Xiao (2018), including 13 items, representative items, such as “companies often improve employee capabilities through cross-departmental cooperation or job rotation,” “companies often improve through cross-departmental cooperation or job rotation” Employee competence.” The reliability coefficient of the scale was 0.878.

### Psychological capital

Measured using the psychological capital scale of Luthans et al. (2005), including 24 items, representative items, such as “I am currently confident about completing my work goals” and “I can always recover from bad emotions quickly when I encounter setbacks at work.” The reliability coefficient of the scale was 0.828.

### Quality of leader-member exchange

Using Liden and Maslyn (1998)‘s leadership member exchange single-dimensional scale measurement, it contained 12 items, representative items, such as “For the boss, even if I have to complete a lot of extra work, I do not mind,” “I am willing to give up better job opportunities for my boss.” According to the method of Harrison and Klein (2007), the average value of LMX of each member in the team was used as the parameter of Quality of leader-member exchange (QLMX). Related research also supported and recognized this method (Homan and Greer, 2013; Huang and Wu, 2019). The reliability coefficient of the scale was 0.899.

### Individual creativity

Used Zhou and George (2001)’s employee innovation performance scale measurement, a total of 6 items, representative items, such as “the subordinate often produces creative ideas and innovative ideas to solve problems,” “the subordinate will Promote your own creativity or innovative ideas step by step.” The reliability coefficient of the scale was 0.891.

### Control variables

Consistent with the existing research (Zhao et al., 2019), this research determined the gender (0 = female; 1 = male) and age (1 = below 30; 2 = 30–39; 3 = 49–49; 4 = 50–59; 5 = over 60 years old;), marriage (1 = married; 2 = unmarried); team tenure (1 = 3–5 years; 2 = 6–10 years; 3 = 11–15 years; 4 = 15 years or more), education (1 = below junior college; 2 = undergraduate; 3 = postgraduate and above) as control variables.

## Results

### Confirmatory factor analysis

Mplus 8.3 was used to perform confirmatory factor analysis on the main variables to evaluate the discriminative validity between the variables. According to Table 1, the four-factor model had a good fit (χ2/*df* = 1.903, RMSEA = 0.042, CFI = 0.961, TLI = 0.950, SRMR = 0.045), which was significantly better than the other three alternative models, indicating that the four-factor model variables had good discrimination validity.

**Table 1:** Fitness indexes of scales.

	Factor composition	χ^2^	df	RMSEA	CFI	TLI	SRMR
Four-factor model	AHRP, PC, QLMX, IC	502.524	243	0.084	0.921	0.905	0.040
Three-factor model	AHRP, PC + QLMX, IC	1070.745	247	0.110	0.876	0.747	0.075
Two-factor model	AHRP, PC + QLMX + IC	1813.500	250	0.254	0.576	0.756	0.154
One-factor model	AHRP + PC + QLMX + IC	2325.204	252	0.354	0.578	0.595	0.135

AHRP, Ambidexterity Human Resource Practice; PC, Psychological Capital; QLMX, Quality of Leader-member exchange; IC, Individual Creativity; “+,” Combined two variables into one factor.

### Data aggregation test

AHRP and QLMX are a team-level (level 2) variable. The questionnaire-answering process needed to be aggregated to the team level because employees would answer the questionnaires. Before aggregation, the group that examined the aggregated variables must be checked for internal consistency (R_wg_) and intra-group correlation coefficients, ICC(1) and ICC(2). When R_wg_ > 0.70, ICC(1) > 0.10, and ICC(2) > 0.70, which indicate that the data aggregation is ideal (Bliese, 2000). One-way analysis of variance showed that the average R_wg_ of AHRP was 0.712, with ICC(1) and ICC(2) of 0.115 and 0.705, respectively; and that the average R_wg_ of QLMX was 0.753, with ICC(1) and ICC(2) of 0.124 and 0.713, respectively. Hence, AHRP and QLMX variable data were satisfactory, met the aggregation requirements, and could be analyzed across levels.

### Common method bias test

Although this study was designed to circumvent the problem of homologous bias in procedures by clarifying the research purpose, emphasizing the confidentiality of information, multi-waves measurement, language specification expression, and differentiated measurement, the same origin bias was still inevitable (Podsakoff et al., 2012). To ensure the rigor of the data, this study used the Harman single factor test method to test the common method bias. The results showed that the unrotated first factor explained 35.817% (<40%) of the variation, and the common factor greater than 1 had 3 eigenvalues. This meant that the problem of homology bias was not serious. Furthermore, this study adopted AMOS 26.0, which used the common method bias as a latent factor to form a five-factor model with the research variables to perform confirmatory factor analysis. The results showed that the four-factor fitting index without the common method bias was: χ^2^/*df* = 2.084, CFI = 0.929, TLI = 0.951, RMSEA = 0.080, and the five-factor model fitting index with the common method bias latent variable was: χ^2^/*df* = 2.062, CFI = 0.931, TLI = 0.965, RMSEA = 0.084, the model fitting index had not been greatly improved (△χ^2^/*df* = 0.022, △CFI = 0.002, △TLI = 0.014, △RMSEA = 0.004). Therefore, there was no serious common method bias problem in this study.

### Descriptive statistics

Table 2 shows the mean, standard deviation, correlation coefficient, and internal consistency coefficient of the main research variables. As shown in Table 2, psychological capital was positively correlated with individual creativity (*r* = 0.496, *p* < 0.01), indicating that the accumulation of employees’ psychological capital promoted individual creativity. Moreover, AHRP was positively correlated with QLMX (*r* = 0.415, *p* < 0.01); AHRP was positively correlated with psychological capital (*r* = 0.428, *p* < 0.01); and AHRP was positively correlated with individual creativity (*r* = 0.419, *p* < 0.01).

**Table 2:** Descriptive statistics and correlation coefficient of variables.

	M	SD	1	2	3	4	5	6
**Individual level**								
Gender	0.471	0.501						
Marriage	1.154	0.604	−0.141^*^					
Age	2.257	0.761	0.033	−0.052^*^				
Team tenure	1.998	1.658	−0.092	0.561^**^	0.021			
Education	2.668	0.353	−0.183^*^	0.313^**^	−0.073	0.371^**^		
Psychological Capital	4.562	0.613	0.074	−0.061^*^		−0.044	−0.154	
Individual Creativity	4.651	0.709	0.069	−0.070		−0.054	−0.151	0.273^*^
**Team level**								
AHRP	5.034	1.354						
QLMX	4.113	0.431						

AHRP, Ambidexterity Human Resource Practice; QLMX, Quality of Leader-member exchange.

* *p* < 0.05.

** *p*<0.01.

### Main effects of AHRP and individual creativity

**Figure 2:**
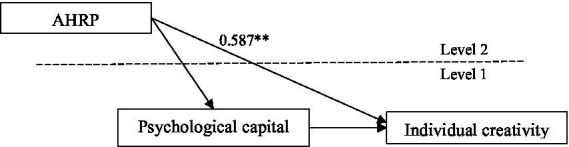
Direct effect coefficient diagram. ^*^p < 0.05, ^**^p < 0.01.

This study used a cross-level analysis to verify the effect of team-level variables on individual-level variables. We used Mplus8.3 (Hayes and Rockwood, 2020) to conduct a 2–1-1″ multi-layer analysis to test the research hypothesis. As shown in Figure 2, the effects of AHRP on individual creativity were significant (*r* = 0.587, *p* < 0.05), which supported H1.

### Mediating effect of psychological capital

**Figure 3:**
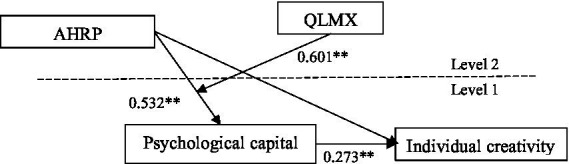
Path coefficient diagram of model. ^*^p < 0.05, ^**^p < 0.01.

To further test the research hypothesis, we drew a path coefficient diagram based on the output of Mplus8.3 (Figure 3). The test results showed that AHRP had significantly positive effects on individual psychological capital (*r* = 0.532, *p* < 0.05); psychological capital had a positive effect on individual creativity (*r* = 0.273, *p* < 0.05); and the mediating effect of psychological capital between AHRP and individual creativity was significant (indirect effect = 0.601, 95% CI [0.388, 0.810], excluding 0). Therefore, H2 was supported.

### Moderating effects of QLMX

**Figure 4:**
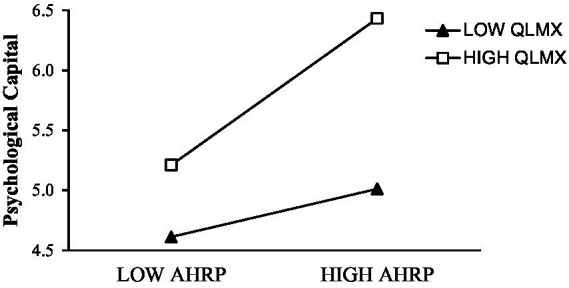
Moderating effect of QLMX between AHRP and psychological capital.

To test the moderating effect of QLMX, we constructed an interactive item of AHRP and QLMX and analyzed psychological capital. The results showed that the interaction terms between AHRP and QLMX had a positive and significant effect on psychological capital (*r* = 0.597, *p* < 0.05). To more intuitively reflect the moderating effect of QLMX, we had further drawn a diagram of the moderating effect of AHRP on psychological capital when QLMX was one standard deviation above and below the average level. As shown in Figure 4 when QLMX was high, the AHRP had more significantly positive effects on psychological capital. Therefore, H3 was verified.

### Moderated mediation effects

To further verify the conditional process model, this study used Monte Carlo repeated sampling test to test the mediating effect of psychological capital (Edwards and Lambert, 2007). The results are shown in Table 3. When QLMX was high, the mediating effect of psychological capital was significant (95% CI [0.559, 0.827], excluding 0). When QLMX was low, the mediating effect of psychological capital was insignificant (95% CI [0.142, 0.485], including 0). However, a difference was observed in the mediating effect between the two levels (Index = 0.571, 95% CI [0.266, 0.879], excluding 0). Therefore, H4 was supported.

**Table 3:** Moderated mediating effect.

QLMX	Mediator variable	Standard error	95% CI	
			LLCI	ULCI
Low level	Psychological capital	0.893^**^	0.559	0.827
High level		0.312^**^	0.142	0.485
Index		0.570^**^	0.266	0.879

**p < 0.01.

## Discussion

### Theoretical contributions

This study explores the mediating effect of AHRP on the individual creativity of employees, reveals the mediating mechanism of employees’ psychological capital and the boundary conditions of QLMX, and draws the following conclusions.

First, AHRP is significantly positively correlated with individual creativity. Creativity requires more energy and resources. When hesitating whether to participate in innovation, employees’ psychological security can inevitably be reduced, in which case they do not propose new ideas and they treat innovative activities negatively. Previous studies mainly discussed the influence of high-performance human resource practice and high-commitment human resource practice on individual creativity (Chang et al., 2014; Chiang et al., 2015). However, in the era of VUCA, only relying on a single human resource practice can no longer meet the dual innovation needs of individuals and organizations. This study also echoes that AHRP can provide resource support for employees’ creativity, and make them enter the value-added spiral, thus delaying or eliminating job burnout or stress caused by resource consumption (Kiazad et al., 2015).

Second, psychological capital mediates the indirect influence of AHRP on individual creativity. In view of the fact that previous studies used work prosperity as the explanation mechanism of AHRP and individual creativity (Chen et al., 2021), this study explored the intermediary mechanism between AHRP and individual creativity from the perspective of psychological capital. The establishment of psychological capital needs to be established in the context of human resources implemented by the organization. AHRP promote the accumulation of individual psychological capital through the two dimensions of commitment and cooperation. Psychological capital is the internal guarantee and driving force of individual behavior, which can effectively promote the generation of individual creativity. Therefore, the effects of AHRP on individual creativity are realized through the accumulation of individual psychological capital.

Third, QLMX plays a moderating role between AHRP and psychological capital. Managers should identify and track the psychological safety status of employees to create a corresponding psychological safety environment. Employees’ psychological safety comes from their cognition of the organizational climate. High QLMX will help employees gain more decision-making power and resources, and will also increase their psychological security (Howell and Hall-Merenda, 1999). Managers actively tolerate employees’ faults and errors while implementing AHRP, provide employees with a relatively safe environment, eliminate their fear of interpersonal risks, and promote innovative behaviors.

Fourth, QLMX moderates the indirect effect of AHRP on individual creativity through psychological capital. Managers should establish diversified interactions with members of the organization, and enhance employees’ perceptions of QLMX. In the management process, leaders should devote themselves to establishing connections with employees in terms of openness, effectiveness, affinity, etc., support employees’ innovation, actively communicate with employees, understand their work needs, and encourage them to seek innovation, give it appropriate autonomy, allow it to try different solutions, accept the failure of its innovation attempt, so as to provide a tolerant management environment for its innovative behavior, so as to improve its psychological security and ensure individual innovation enthusiasm.

### Practical implications

This study provides organization managers the following practical enlightenment.

First, organizations need to implement AHRP. As the external environment becomes more and more complex, organizations usually face different management paradoxes, the most common of which is to pursue the balance between utilization innovation and exploratory innovation. AHRP help the team integrate internal and external resources, strengthen internal coordination and cooperation, and adapt to external needs. Their effective implementation can bring a series of benign effects to the team and the organization. Specifically, in each team, an enterprise can implement a scientific AHRP configuration based on its own actual situation to help organizational members enhance their individual creativity and achieve organizational innovation and development.

Second, the psychological capital of organization members should be increased. Psychological capital is a positive psychological resource. Previous studies have shown that it has a positive relationship with work attitude and behavioral variables such as job satisfaction (Zhang et al., 2021), organizational commitment (Tang et al., 2019) and organizational citizenship behavior (Hu et al., 2018). The results of this study also confirm the positive role of psychological capital. Therefore, in the practice of enterprise management, managers can cultivate and develop employees’ psychological capital by measuring the “stock” of employees’ psychological capital, or encourage employees to carry out positive self-cultivation to improve their psychological capital.

Third, QLMX in the organization should be improved. Organizations need to pay certain attention to the establishment of a stable and active exchange relationship between leaders and members, improve communication methods, and expand communication channels. Harmonious interpersonal communication is a key factor in promoting organizational cohesion. The high-quality interpersonal relationship among organization members can promote knowledge sharing between team members. In addition, leaders must actively formulate a fair and open competition mechanism, pay attention to employees’ investment in innovation and their enthusiasm for work and study to ensure that employees achieve a better state of psychological safety, stimulate their enthusiasm for work, and increase their willingness to participate in innovation.

### Limitations and future directions

This study is based on social exchange theory, through multi-agent, multi-temporal, and multi-level follow-up investigations. It has explored the mediating mechanism of AHRP’s moderation of individual creativity and obtained many useful conclusions and enlightenments. Nevertheless, it still has the following shortcomings.

First, data collection. Although multiple time-point data are used to strengthen the causal consistency between variables in terms of time to avoid problems, such as homology variance, non-longitudinal research is difficult to objectively reflect the causal relationship between variables. Therefore, future works can adopt longitudinal, experimental, and objective research. Moreover, evaluation method presents the causal relationship between variables in a true and objective manner. In addition, the practice of psychological capital and AHRP for data collection is performed in a self-evaluation method, which will inevitably have a praise effect on organizational evaluation. Therefore, in-depth interviews can be used for future data collection to improve the objectivity of variable measurement.

Second, sample selection. The sample data of this study are all from China and are influenced by Chinese traditional culture. The concept of “home and everything is prosperous” affects the status of the family in people’s minds, and the “circle culture” affects the interpersonal relationship within the organization. Therefore, this study only considers the Chinese context and has certain cultural limitations, which affect the universality of the conclusions. Therefore, future works can be integrated into different cultural contexts to expand the universality of research conclusions.

## Conclusion

Organizations implement effective AHRP, which can improve employees’ psychological capital, thus helping to enhance employees’ creativity. According to the research results, we discuss as follows:

First of all, AHRP has a positive and significant impact on individual creativity. The data results in Figure 2 supported the direct relationship between AHRP and individual creativity. On the one hand, the research results echo the existing research on human resource practice to enhance individual creativity (Han and Yang, 2011). When employees perceive their appreciation, recognition and investment in human resource practices, they can enhance their individual’s perception of work safety (Hu et al., 2017), thereby stimulating innovation motivation (Han and Yang, 2011), and enhancing individual creativity (Bledow et al., 2009). On the other hand, the research conclusion points out that AHRP has an impact on individual creativity by helping to improve employees’ psychological security, echoing Xiao (2018)’s conclusions on the role of human resources and individual psychological perception, and testing the existing research conclusions.

Second, psychological capital mediates the indirect relationship between AHRP and individual creativity. We tested and confirmed the mediating effect of psychological capital through Monte Carlo repeated sampling method. As a mediating between organizational practice and individual behavior, psychological capital is not only a further test of resource conservation theory, it also echoes the research conclusions of Luthans et al. (2005) that changes in psychological capital affect individual behavioral performance. The implementation of AHRP can have a positive impact on the accumulation of individual psychological capital, enhance individual self-efficacy, and then affect individual creativity. AHRP echoes Hu et al. (2017)‘s research initiative of “organizational diversity promotes individual innovation,” and clarifies the internal mechanism of the trickle-down effect of human resource practice.

Finally, QLMX moderates the positive effect of AHRP on psychological capital. The moderating effect diagram in Figure 4 clearly showed the promoting effect of high QLMX on the relationship between AHRP and psychological capital. In the context of “circle culture,” the quality of interpersonal relationships within the organization has to be considered. “Circle culture” brings about an imbalance in the distribution of resources (Zhao et al., 2019). This kind of resources includes not only material resources, but also emotional and psychological resources (Luthans et al., 2005). The research conclusions further test the theory of social exchange in the context of China’s “circle culture.”

## Data availability statement

The raw data supporting the conclusions of this article will be made available by the authors, without undue reservation.

## Ethics statement

Ethical review and approval was not required for the study on human participants in accordance with the local legislation and institutional requirements. The authors declare that they strictly adhered to the APA guidelines on ethical research practices. Written informed consent for participation was not required for this study in accordance with the national legislation and the institutional requirements. The patients/participants provided their online informed consent to participate in this study, which stated the voluntary nature of participation, and assurance of confidentiality and anonymity.

## Author contributions

All authors listed have made a substantial, direct, and intellectual contribution to the work and approved it for publication.

## Funding

This study received funding from Fundamental Research Funds for the Central Universities (2021-zy-011).

## Conflict of interest

The authors declare that the research was conducted in the absence of any commercial or financial relationships that could be construed as a potential conflict of interest.

## Publisher’s note

All claims expressed in this article are solely those of the authors and do not necessarily represent those of their affiliated organizations, or those of the publisher, the editors and the reviewers. Any product that may be evaluated in this article, or claim that may be made by its manufacturer, is not guaranteed or endorsed by the publisher.
